# Evaluation of cardiovascular autonomic dysfunction in symptomatic post-COVID-19 patients using the heart rate variability (HRV) and detection of subtle LV dysfunction using 2D global longitudinal strain (GLS)

**DOI:** 10.1007/s10554-023-02915-w

**Published:** 2023-09-02

**Authors:** Amira Nour, Mirna Fouad, Zeinab Abdel Salam

**Affiliations:** Cardiology department, Ain Shams University, Cairo, Egypt

**Keywords:** Symptomatic post-COVID-19, Autonomic dysfunction, Global longitudinal strain, Heart rate variability

## Abstract

**Aim:**

The COVID-19 disease primarily affects the respiratory system; however, cardiac involvement has been documented in the acute phase. We aimed to evaluate the cardiac autonomic function and subtle left ventricular dysfunction in those subjects recovered from mild to moderate acute COVID-19 patients but still symptomatic.

**Methods and results:**

The study group was composed of 50 subjects with confirmed mild to moderate COVID-19. All subjects underwent routine 2D echocardiography assessment in addition to 2D speckle tracking and 24 h Holter monitoring for HRV analysis. The mean age of the study population was 42 ± 18 years; symptoms were reported as follows 27 (54%) had dyspnoea, 17 (34%) had palpitation, and 7 (14%) had dizziness. Time domain parameters Standard Deviation of NN intervals (SDNN), Standard Deviation of the Average NN intervals for each 5 min segment of a 24 h HRV recording (SDANN), and Root Mean Square of Successive RR interval Differences (rMSSD) were diminished with mean SDNN value being markedly impaired in 12 (24%) patients, while frequency domain parameters as assessed by the ratio of the Low-Frequency band power to the High-Frequency band power (LF/HF) with the mean of 1.837 with 8% of the patients being impaired. SDNN was significantly reduced in patients with impaired global longitudinal strain (p 0.000). The global longitudinal strain was diminished in 10 patients (20%); also, 80% of the patients with impaired GLS had decreased SDNN.

**Conclusion:**

Our study targeted patients experiencing prolonged symptoms after COVID-19 illness. We detected a high incidence of GLS impairment using Speckle Tracking Echocardiography (STE) and a significant prevalence of diminished HRV. HRV (especially SDNN) and GLS were found to be significantly correlated.

**Graphical abstract:**

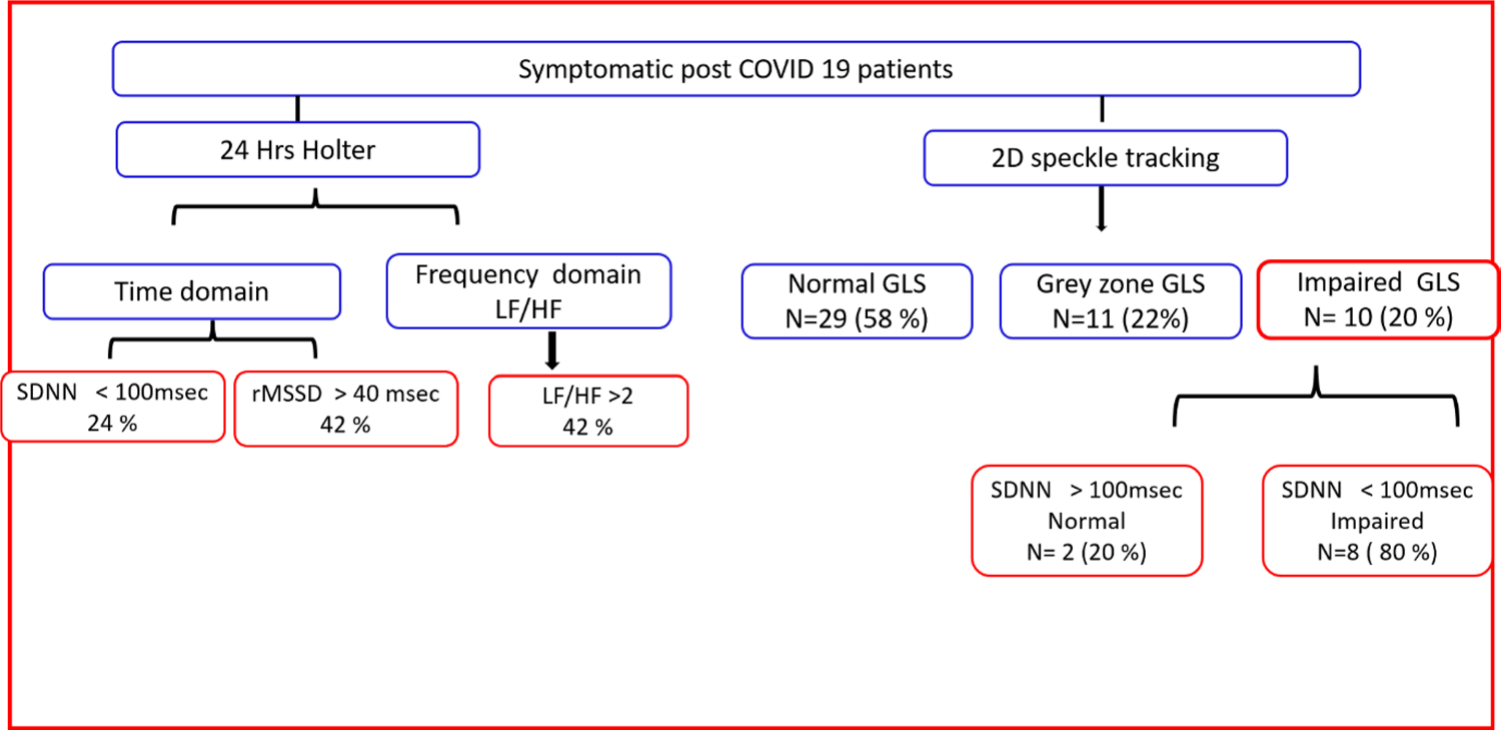

## Introduction

Although the respiratory tract is the primary target of the Coronavirus disease 2019 (COVID-19), cardiovascular involvement has been documented in the acute stages of the disease [1]. cardiovascular involvement has been in the form of myocarditis, myocardial ischemia, heart failure, and thromboembolic manifestation [2]. Many patients recover entirely from COVID-19; however, some patients still complain of diverse symptoms such as palpitations, dyspnea on exertion, fatigue, dizziness, and headache, which goes with altered autonomic functions and underlying cardiac affection [3]. The acute manifestation of COVID-19 has been widely studied and researched; however, the post-COVID-19 sequela needs further investigation.

### HRV indices

It has been suggested that this virus may affect the brain cells and the hypothalamic pituitary adrenal axis, which may cause altered autonomic functions [4]. Since then, we found that the heart rate variability with its indices has been a reliable and validated method for quantitatively assessing the sympathetic and parasympathetic nervous systems. It is also well known that decreased HRV correlates with an increased risk of cardiovascular death and all-cause mortality [5]. So, we aimed to study the HRV and the autonomic functions in recovered COVID-19 patients who had mild to moderate acute COVID-19 infection within 12 weeks from the active infection since most studies studied the autonomic dysfunction in the period of acute infection.

### Echocardiographic evaluation

Since myocardial injury secondary to COVID-19 is associated with poor prognosis during acute COVID-19 infection [6], it is unclear whether this myocardial insult may have short-term or long-term sequelae, especially in patients who continue to be symptomatic despite the clearance of the viral infection [7]. Cardiac MRI revealed that up to 90% of the patients continue to have myocardial edema and inflammation after COVID-infection [8]. 2D-STE can detect subclinical myocardial dysfunction earlier than the conventional 2D echo in symptomatic post-COVID-19 patients. So, we aimed to study the subtle LV dysfunction in those patients, its correlation with the symptoms, and HRV indices as assessed by the 24-h Holter monitoring.

## Patients and methods

We included fifty consecutive subjects between 17 and 82 years of age who were symptomatic after three weeks up to three months of experiencing mild to moderate COVID-19 illness, according to the National Institutes of Health, United States (NIH). Patients enrolled were coming for follow-up clinic visits at Ain Shams University hospitals between June 2021–June 2022. [10].

COVID-19 diagnosis was confirmed using real-time reverse-transcriptase polymerase chain reaction assays on nasopharyngeal swabs or typical symptoms of COVID-19 in addition to high/very high suspicion (CO-RADS 4 or 5) of COVID-19 on High-Resolution Computed Tomography of the chest (HRCT Chest), or both [9].

### Inclusion criteria

1. Successive 50 symptomatic patients presented after mild to moderate COVID-19 illness, according to the NIH [10].
2. The selected symptoms for inclusion were tiredness, fatigue, dizziness, palpitations, chest pain, and shortness of breath.

### Exclusion criteria

1. Patients with a history of severe or critical COVID-19 illness according to the NIH [10] or hospitalized in ICU, multiple organ failure, or needed domiciliary oxygen for the index COVID-19 infection or with active infection; within 14 days from symptom onset or PCR positive result.
2. Those with a history of myocardial infarction, previous PCI, or coronary artery bypass graft (CABG),
3. Those with known lung disease, LVEF < 55%, or segmental wall motion abnormalities.
4. Those with arrhythmias, LBBB, RBBB, or cardiac pacemakers.
5. Any known type of cardiomyopathies or valvular heart diseases.
6. Those with thyroid dysfunction and chronic renal disease (GFR < 60 mL/min/1.73 m^2^), malignancy, or use of cardiotoxic-related medications.
7. Patients with poor 2D image quality.

After written informed consent from the subjects or their guardians and approval of Ain Shams ethical committee was obtained.

Detailed history taking and full clinical examination were done, followed by 2D conventional echocardiography and 24-h Holter monitoring.

#### 2D Echocardiographic image acquisition and analysis of the LV-GLS [11]

Transthoracic 2D echocardiography was done by an experienced cardiologist having the patient in a supine position using M5Sc-D [1.4–4.6 MHz] probe with a “GE Vivid E95” echocardiography machine.

Standard images were obtained in the parasternal (long- and short-axis views) and apical (2, 3,4, and 5-chamber images) views.

Standard 2D and color Doppler data were saved in cine-loop format and triggered to the QRS complex.

M-mode, 2D, and pulsed and continuous Doppler flow across the different heart valves in all the standard views were done according to the American Society of Echocardiography recommendations.

The following measurements were focused on: Assessment of LV systolic function by M mode, MAPSE (Mitral Annular Plane Systolic Excursion), and modified Simpson's Method (the endocardium was traced in the end diastole and end systole in the apical four chambers and two chambers views), LA/Ao dimension by M-mode, Wall motion abnormality (any patient with resting segmental wall motion abnormalities was excluded from the study), assessment of LV diastolic function using pulsed-wave Doppler and TDI on the mitral annulus.

Digital loops of multiple ECG- gated entire cardiac cycles of the LV were acquired from apical 2-, 3-, and 4-chambers.

Peak Global longitudinal strain (GLS) was analyzed using computer software for tissue tracking; EchoPAC Dimension [12.0, General Electrics (GE) Medical Systems GmbH, Germany].

**Fig. 1:**
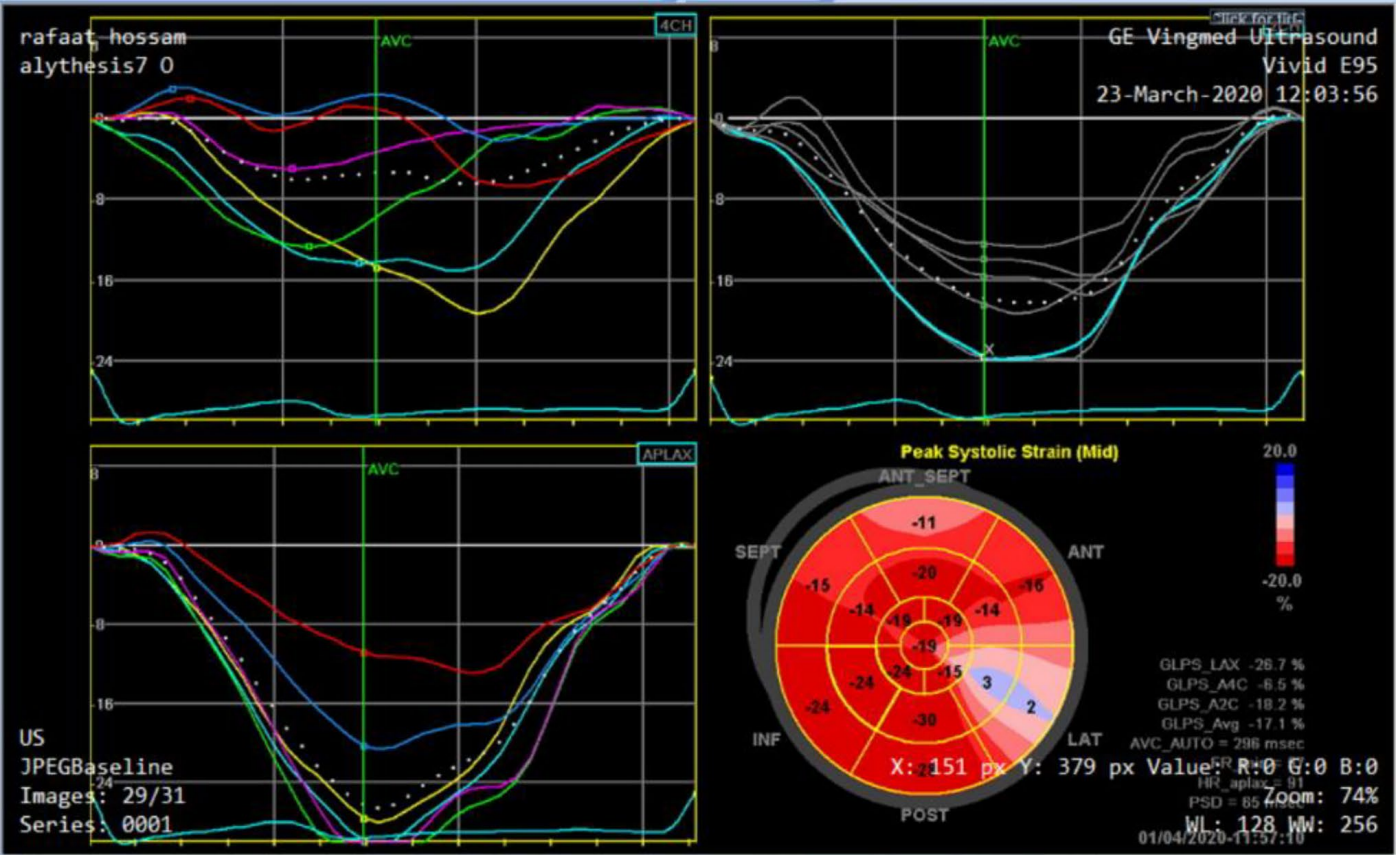
From subject No (9) showing DICOM images presenting curves of the longitudinal strain of LV at the apical 4,3 and 2 chambers views with an estimation of the global longitudinal strain and bull eye

This software recorded peak systolic longitudinal strain for each myocardial segment. The strain values for all the segments are recorded and averaged to obtain the global longitudinal strain (GLS). A topographic representation of the regional and global longitudinal strain of all 17 analyzed segments (Bull's eye configuration) was then automatically generated, Fig. 1.

According to the latest American Echocardiography Association guidelines, for LVGLS, above − 18% is defined as normal, − 16% − 18% as gray zone, and under − 16% as diminished. [11].

Twenty-seven patients underwent LVEF assessment before the COVID infection, using 2D and M-mode, and all had LVEF > 55%.

#### Holter monitoring & HRV parameters

Twenty-four hours of Holter monitoring was performed on all patients on the same day as the echocardiography, using the “CONTEC TLC9803” Dynamic 3-channel ECG Holter monitor. HRV was evaluated using time-domain and frequency-domain parameters.

Time-domain parameters included were: SDNN (Standard Deviation of NN intervals), SDANN (Standard Deviation of Average of NN intervals), and RMSSD (Root Mean Square of Successive Differences between adjacent NNs), all measured in milliseconds (msec).

Frequency-domain parameters included were: LF (Low Frequency) measured in squared milliseconds (msec " HRV data analysis in the study population :"), HF (High Frequency) measured in msec "HRV data analysis in the study population :", and LF/HF ratio.

The SDNN, SDANN, and SDNN Index are obtained from long-term records and represent the sympathetic and parasympathetic activity, but they do not allow distinguishing when changes in HRV are due to increased sympathetic tone or the withdrawal of vagal tone. The rMSSD represents the parasympathetic activity as they are found from the analysis of adjacent RR intervals.

SDNN values predict both morbidity and mortality. Based on 24 h monitoring, patients with SDNN values below 50 ms are classified as unhealthy, 50–100 ms have compromised health, and those above 100 ms are healthy [12]. RMSSD > 40 indicates parasympathetic predominance [13].

On the other hand, the High-Frequency component (HF) corresponds to respiratory modulation and is an indicator of the performance of the parasympathetic vagal innervation in the heart. In comparison, the Low-Frequency component (LF) is due to the combined action of the vagal and sympathetic components on the heart, with a predominance of the sympathetic ones.

The LF/HF ratio reflects the absolute and relative changes between the sympathetic and parasympathetic components of the autonomic nervous system by characterizing the sympathetic vagal balance on the heart.

LF/HF below 1.5 indicates parasympathetic predominance, while above 2 indicates sympathetic predominance [12, 13].

24-h Holter data were also evaluated for arrhythmias, including atrial fibrillation, atrial flutter, supraventricular tachycardia, frequent premature ventricular contractions (defined as ≥ 10% premature ventricular contractions on 24-h Holter recording), ventricular tachycardia, ventricular fibrillation, and atrioventricular block (second degree or higher).

Most patients were not on specific medications; however, 12 patients were hypertensive, taking angiotensin-converting enzyme inhibitor (ACEI) or angiotensin receptor blocker (ARB).

## Statistics

Data were collected, revised, coded, and entered into the Statistical Package for Social Science (IBM SPSS) version 23. The quantitative data were presented as mean, standard deviations, and ranges when parametric and median, and inter-quartile range (IQR) when data was found non-parametric. Also, qualitative variables were presented as numbers and percentages.

The comparison between groups regarding qualitative data was done using the Chi-square test and/or Fisher exact test when the expected count in any cell was less than 5.

*One way ANOVA* (analysis of variance) *test* was used to compare more than two groups regarding quantitative variables. *Spearman correlation coefficients* were used to assess the correlation between two quantitative parameters in the same group. *The receiver operating characteristic curve (ROC)* was used to determine the best cut-off point with its sensitivity, specificity, positive predictive value, negative predictive value, and area under the curve (AUC) of the studied marker.

## Results

Our study included a total of 50 patients with a mean age of 42, and 76% were male as opposed to 24% females, where the most common symptom was fatigue (82%) followed by Dyspnea (54%). Table 1 includes demographic data, cardiovascular risk factors, post-COVID symptoms, and echocardiographic data, including global longitudinal strain in the study population.

**Table 1:** Demographic data for the study population, including echocardiographic and Holter parameters

	Number
Age	42.06 ± 18.51
Male sex	38 (76%)
Risk factors	
Dm	11 (22.0%)
HTN	12 (24.0%)
Smoking	8 (16.0%)
Dyslipidemia	4 (8.0%)
Post covid symptoms	
Chest pain	8 (16%)
Dyspnea	27 (54%)
Palpitations	17 (34%)
Dizziness	7 (14%)
Fatigue	41 (82%)
Echocardiographic data	Mean ± SD
2D EF %	62.62 ± 5.43
Modified Simpson's	62.26 ± 4.98
MAPSE in cm	1.37 ± 0.25
TAPSE in cm	1.90 ± 0.20
LVEDD in mm	47.60 ± 2.62
LVESD in mm	27.62 ± 3.90
Aortic diameter(mm)	24.50 ± 2.05
LA diameter (mm)	32.34 ± 3.94
Diastolic dysfunction	
No DD	32 (64.0%)
Grade I	10 (20.0%)
Grade II	8 (16.0%)
Grade III	0 (0.0%)
GLS in %	− 18.07 ± 2.48
Normal >—18%	29 (58%)
Grey zone − 16 to − 18%	11 (22%)
Diminished < − 16%	10 (20%)
Holter parameters	
Max HR	115.90 ± 16.86
Min HR	57.64 ± 7.05
SDNN	117.47 ± 24.66
SDANN	102.24 ± 27
**r**MSSD (median) (IQR)	30.9 (24.3–46.1)
LF (median) (IQR)	744.6 (326.6–1124)
HF (median) (IQR)	429 (161–682.5)
LF/HF (median) (IQR)	1.837 (0.8737–2.8999)

### Left ventricle Global Longitudinal Strain by Speckle Tracking Echocardiography and its correlation with the Holter parameters:

**Fig. 2:**
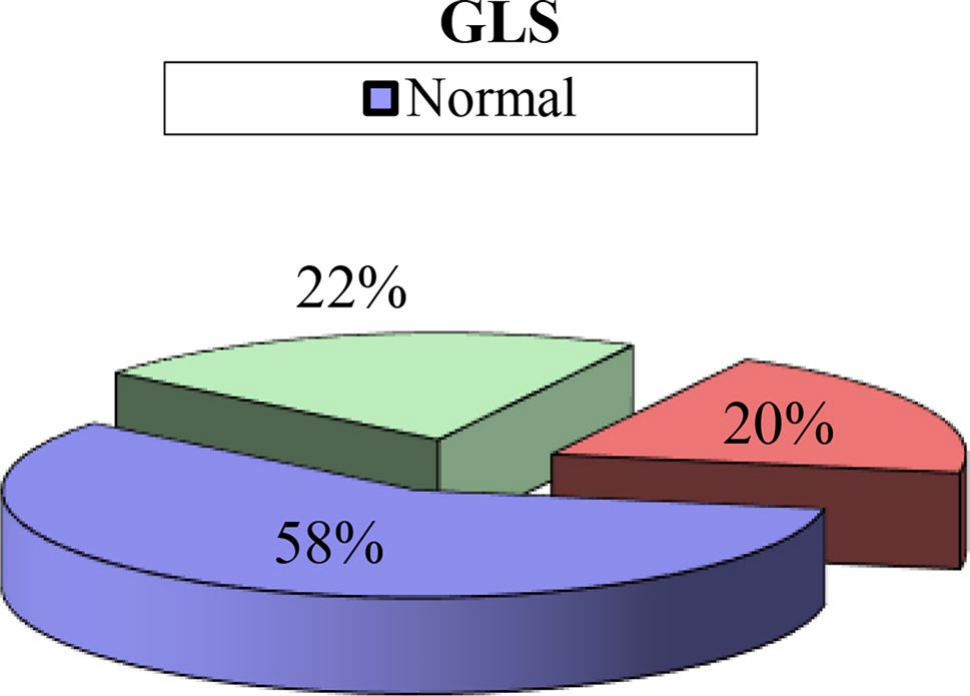
Global longitudinal strain among the study population

Twenty-nine of our patients (58%) had normal GLS, eleven patients (22%) were in the grey zone with a GLS between − 18 to–16%, ten patients (20%) had a diminished GLS below -16% as shown in Fig. 2.

Regarding post-COVID symptoms, Only dyspnea was found to be statistically significant in patients with impaired GLS (P value = 0.029), also GLS value was found to be significantly lower in the elderly patients (p-value = 0.001), diabetics (p-value 0.003) and hypertensive patients (p-value 0.002) as shown in Table 2.

**Table 2:** Relation of GLS with demographic, CVS risk factors, presenting symptoms, and Holter parameters

	GLS			Test value	P-value	Sig
	Normal	Gray zone	Diminished			
	No. = 29	No. = 11	No. = 10			
Demographic data:						
Age				37.971•	0.001	HS
Mean ± SD	32.66 ± 10.92	41.45 ± 16.32	70.00 ± 6.83			
Range	18–60	26–66	64–80			
Gender				1.782*	0.410	NS
Male	23 (79.3%)	9 (81.8%)	6 (60.0%)			
Female	6 (20.7%)	2 (18.2%)	4 (40.0%)			
CVS risk factors						
DM				27.961*	0.003	HS
No	29 (100.0%)	8 (72.7%)	2 (20.0%)			
Yes	0 (0.0%)	3 (27.3%)	8 (80.0%)			
HTN				22.048*	0.001	HS
No	27 (93.1%)	9 (81.8%)	2 (20.0%)			
Yes	2 (6.9%)	2 (18.2%)	8 (80.0%)			
Smoker				0.263*	0.877	NS
No	25 (86.2%)	9 (81.8%)	8 (80.0%)			
Yes	4 (13.8%)	2 18.2%)	2 (20.0%)			
Dyslipidaemia				3.148*	0.207	NS
No	25 (86.2%)	11 (100.0%)	10 (100.0%)			
Yes	4 (13.8%)	0 (0.0%)	0 (0.0%)			
Dyspnoea				7.078*	0.029	s
No	17 (58.6%)	5 (45.5%)	1 (10.0%)			
Yes	12 (41.4%)	6 (54.5%)	9 (90.0%)			
Holter parameters						
Max HR						
Mean ± SD	118.66 ± 16.67	118.00 ± 15.66	105.60 ± 16.20	2.481•	0.095	NS
Range	88 – 148	89 – 134	90 – 127			
Min HR				4.163•	0.022	S
Mean ± SD	56.03 ± 6.43	57.00 ± 6.03	63.00 ± 7.80			
Range	43–67	49–66	55–76			
SDNN				13.170•	0.001	HS
Mean ± SD	122.19 ± 22.98	130.86 ± 15.92	89.04 ± 14.05			
Range	71.8–154.1	100.4–144.3	65–104.3			
SDNN				22.323*	0.001	HS
Normal	25 (86.2%)	11 (100.0%)	2 (20.0%)			
Impaired	4 (13.8%)	0 (0.0%)	8 (80.0%)			
SDANN				9.817•	0.001	HS
Mean ± SD	101.94 ± 28.59	124.12 ± 12.33	79.04 ± 10.98			
Range	56.7–142.6	100.2–135.9	61.5–93			
rMSSD				2.504‡	0.286	NS
Median (IQR)	32.1 (27.4–49)	27.9 (23.9–41.1)	24.3 (22.5–44.9)			
Range	17.6–190.6	22.6–49.1	18.3–67.1			
LF				21.574‡	0.110	NS
Median (IQR)	744.6 (356 – 1135.7)	1001.7 (921.8 – 1147.2)	173.7 (92 – 326.4)			
Range	111.1 – 1611.5	765 – 1206	24 – 331			
HF				10.475‡	0.296	NS
Median (IQR)	429 (158.8–845.8)	571.4 (489.3–850)	161 (75.7–190.7)			
Range	40.7–1963.6	316.9–987.5	28.2–543.8			
LF/HF				5.596‡	0.061	NS
Median (IQR)	2.3194 (0.8737–4.37)	1.8839 (1.0015–2.0077)	0.8511 (0.6087–1.7116)			
Range	0.1223–38.5504	0.9–3.1609	0.5714–2.2946			
LF/HF				5.621*	0.229	NS
< 1.5	10 (34.5%)	3 (27.3%)	6 (60.0%)			
1.5–2	4 (13.8%)	4 (36.4%)	2 (20.0%)			
> 2	15 (51.7%)	4 (36.4%)	2 (20.0%)			

P-value > 0.05: Non significant (NS); P-value < 0.05: Significant (S); P-value < 0.01: highly significant (HS)

^*^Chi-square test; •Independent t-test; ‡Mann Whitney test

**Fig. 3:**
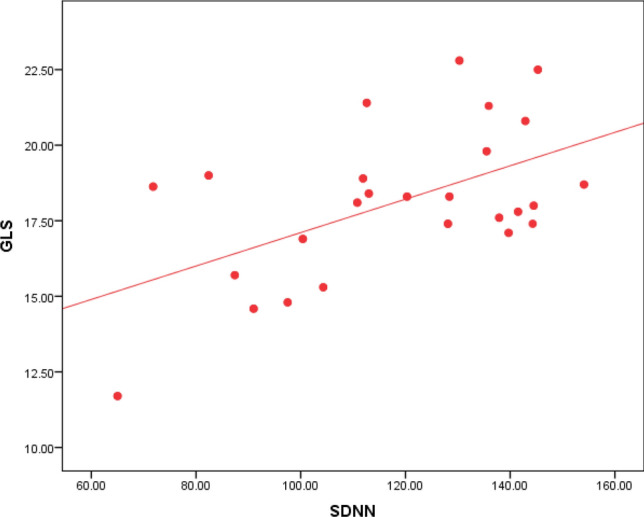
Shows a positive linear correlation between GLS and SDNN with r 0.415 and p-value of 0.003

**Fig. 4:**
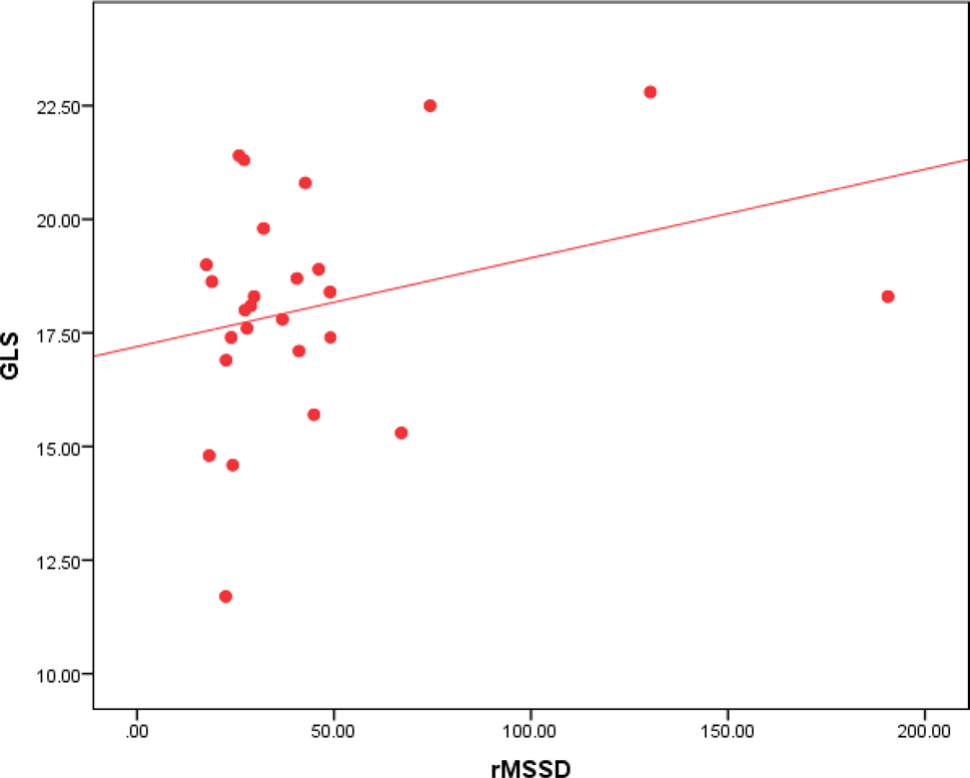
Shows a positive linear correlation between GLS and rMSSD with r 0.306 and p-value of 0.031

**Fig. 5:**
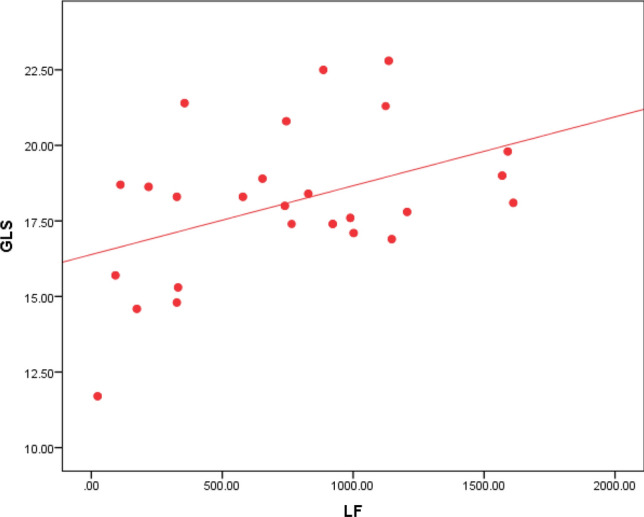
Shows a positive linear correlation between GLS and LF with r 0.0350 and p-value of 0.013 between GLS and LF/HF with r 0.309 and p-value of 0.029

**Fig. 6:**
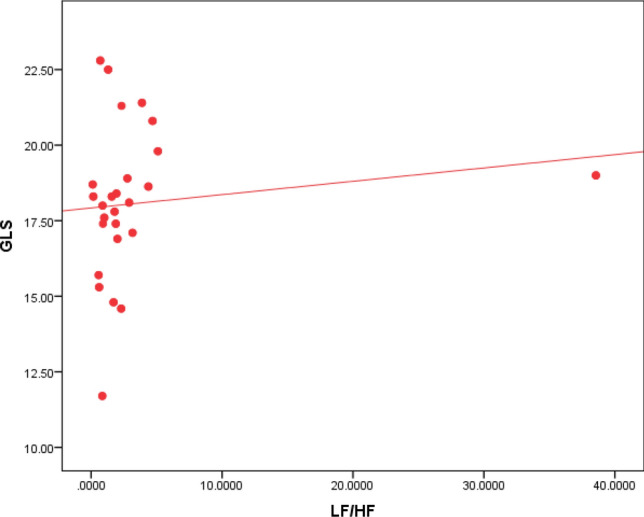
Shows a positive linear correlation

Regarding Holter parameters, there is a statistical significance between SDNN, SDANN, and GLS, as 80% of the patients with impaired GLS have decreased SDNN, and 79% of the patients with impaired GLS have decreased SDANN (Table 2). The ROC curve analysis failed to point out a significant relation between LVGLS and the other HRV parameters except for SDNN and SDANN. However, using Spearman's correlation coefficient analysis, GLS values were plotted against the values of all HRV parameters. GLS was positively correlated to rMSSD, LF, and LF/HF ratio with p values 0.031, 0.013, and 0.029, respectively (Figs. 3, 4 & 5). GLS showed a positive correlation with SDNN with a p-value of 0.003 (Fig. 6).

P-value > 0.05: Non significant (NS); P-value < 0.05: Significant (S); P-value < 0.01: highly significant (HS) *: Chi-square test; •: One Way ANOVA test; ‡: Kruskal Wallis test.

### HRV indices and its correlation with risk factors, symptoms, and global longitudinal strain

**Fig. 7:**
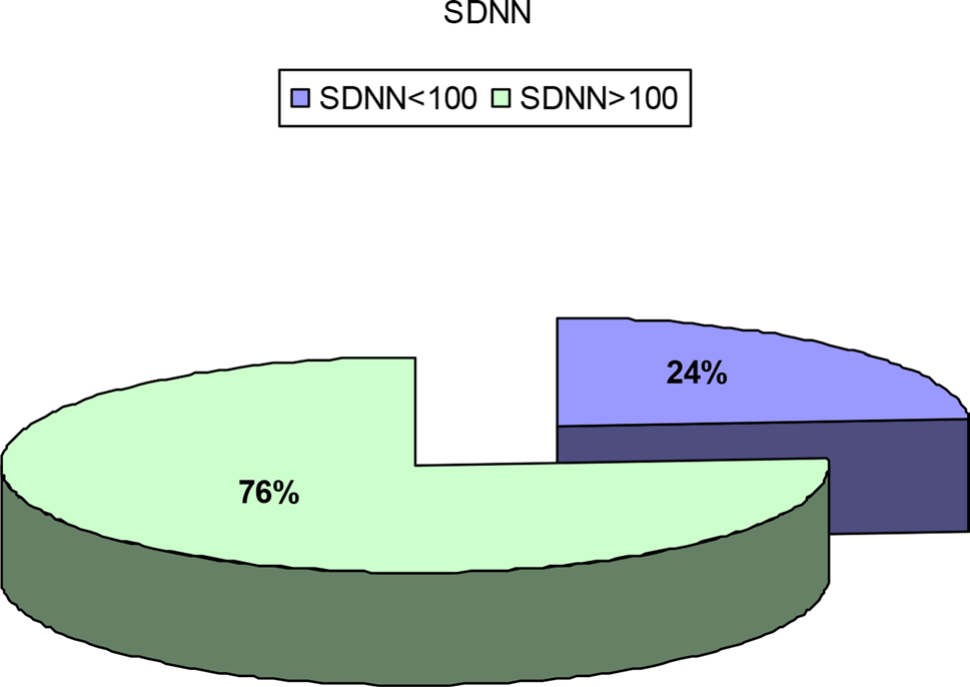
Shows SDNN distribution in the study population

SDNN: SDNN was below 100 ms in 12 patients (24%), while 38 had an SDNN above 100. No patients were reported to have an SDNN below 50; therefore, there were no patients with severely impaired HRV, as illustrated in Fig. 7**.**

In the study population, SDNN was significantly impaired in the elderly and diabetic patients, p values 0.001 and 0.007, respectively. There was a statistically significant increase in the percentage of female patients with SDNN < 100 ms (50%) than with SDNN > 100 ms (only 15.8%). The correlation of presenting post-COVID symptoms to HRV measured by SDNN was highly significant to palpitation as the presenting symptom. However, no other symptom was correlated to SDNN, as shown in Table 3.

**Table 3:** Relation of SDNN with risk factors, symptoms, and echocardiographic data

		SDNN		Test value	P- value	Sig
		> 100 ms	< 100 ms			
		No. = 38 (76%)	No. = 12 (24%)			
Age	Mean ± SD	37.18 ± 15.33	57.50 ± 19.87	-3.723•	0.001	HS
	Range	18–71	24–80			
Gender	Male	32 (84.2%)	6 (50.0%)	5.852*	0.016	S
	Female	6 (15.8%)	6 (50.0%)			
*CVS risk factors: -*						
DM	No	33 (86.8%)	6 (50.0%)	7.214*	0.007	HS
	Yes	5 (13.2%)	6 (50.0%)			
HTN	No	32 (84.2%)	6 (50.0%)	5.852*	0.016	S
	Yes	6 (15.8%)	6 (50.0%)			
Smoker	No	32 (84.2%)	10 (83.3%)	0.005*	0.942	NS
	Yes	6 (15.8%)	2 (16.7%)			
Dyslipidaemia	No	36 (94.7%)	10 (83.3%)	1.611*	0.204	NS
	Yes	2 (5.3%)	2 (16.7%)			
Palpitations	No Yes	29 (76.3%) 9 (23.7%)	4 (33.3%) 8 (66.7%)	7.509*	0.006	HS
Holter parameters						
2D EF	Mean ± SD	63.29 ± 5.41	60.50 ± 5.14	1.575•	0.122	NS
	Range	50 – 72	55 – 70			
Simpson's	Mean ± SD	62.87 ± 5.14	60.33 ± 4.03	1.561•	0.125	NS
	Range	52 – 70	54 – 66			
DD	No DD	28 (73.7%)	4 (33.3%)	9.430*	0.912	NS
	Grade I Grade II	4 (10.5%) 6 (15.8%)	6 (50.0%) 2 (16.7%)			
MAPSE	Mean ± SD	1.41 ± 0.22	1.25 ± 0.30	2.034•	0.471	NS
	Range	1 – 1.9	0.8 – 1.5			
TAPSE	Mean ± SD Range	1.96 ± 0.19 1.7 – 2.4	1.73 ± 0.10 1.6 – 1.9	3.816•	0.973	NS
GLS	Mean ± SD	-18.81 ± 1.95	-15.74 ± 2.61	-4.379•	0.001	HS
	Range	-15.3 – -22.8	-11.7 – -19			
GLS	Normal	25 (65.8%)	4 (33.3%)	22.323*	0.001	HS
	Gray zone	11 (28.9%)	0 (0.0%)			
	Diminished	2 (5.3%)	8 (66.7%)			

P-value > 0.05: Non significant (NS); P-value < 0.05: Significant (S); P-value < 0.01: highly significant (HS)

^*^Chi-square test; •Independent t-test; ‡Mann Whitney test

**Fig. 8:**
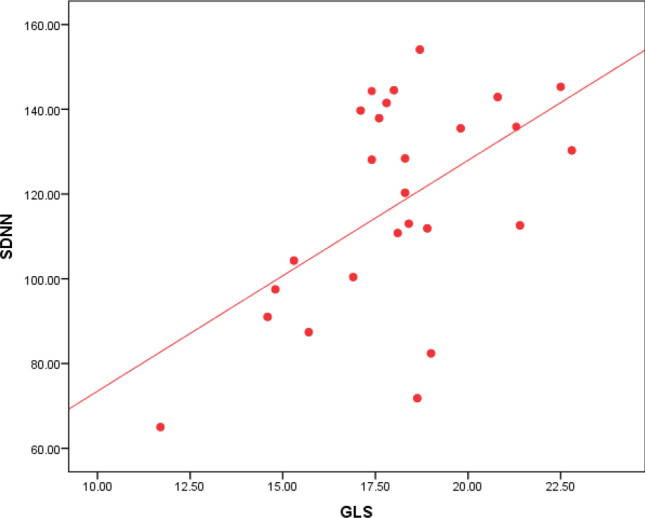
Shows a positive linear correlation between SDNN and GLS with r 0.415 and p-value of 0.003

Analysis of SDNN and echocardiographic data showed a highly significant relation between impaired SDNN and impaired GLS with a p-value of 0.001, as shown in (Table 3). SDNN values were also plotted against GLS values showing a positive correlation between both, as shown in Fig. 8. Other echocardiographic parameters were not significantly related to SDNN.

While analysis of rMSSD with a value below 40 ms was not significantly correlated with any of the echocardiographic data.

**Fig. 9:**
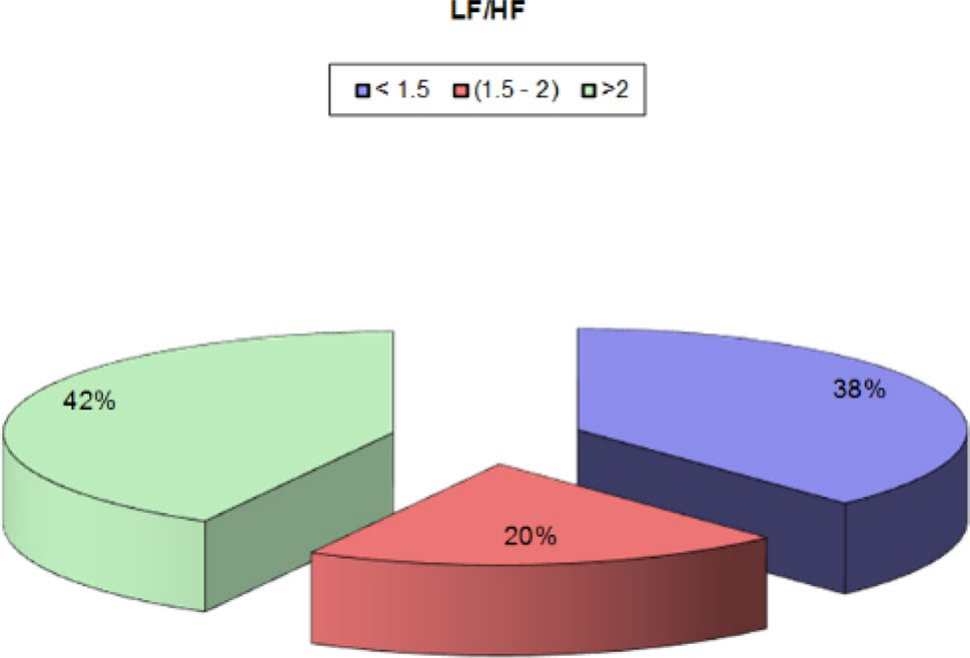
LF/HF ratio among the study population

**Fig. 10:**
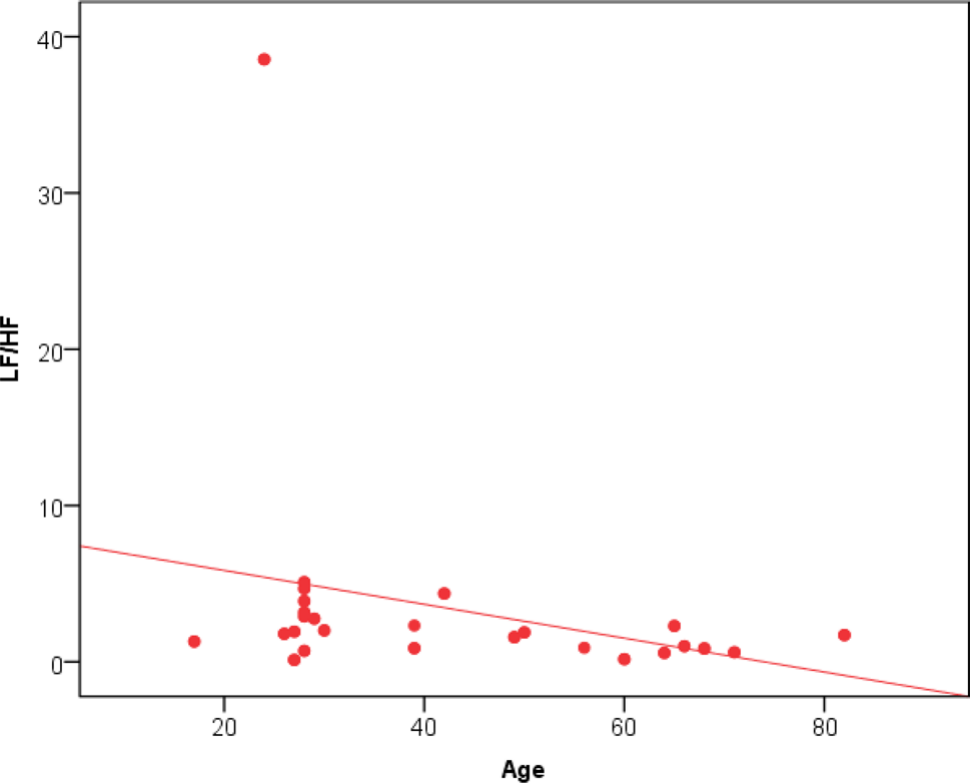
Shows a negative linear correlation between LF/HF and age with r -0.309 and p-value 0.006

**Fig. 11:**
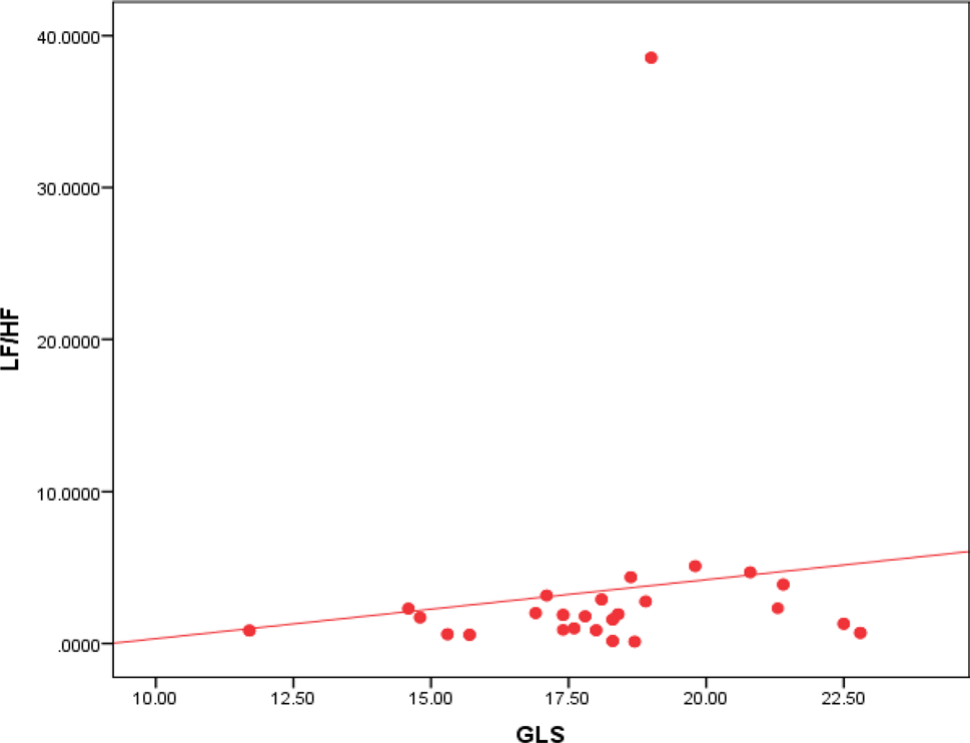
Shows a positive linear correlation between LF/HF and GLS with r 0.309 and p-value 0.029

Analysis of the LF/HF ratio showed that 38% of the study population had LF/HF ratio below 1.5, as shown in Fig. 9. There was no association between LF/HF and post-COVID symptoms. However, DM and increased age were statistically significant as none of the diabetic patients had an LF/HF ratio > 2 with a p-value of 0.006. Also, there was a negative linear correlation between the LF/HF ratio and age, i.e., the older the age, the lower the LF/HF ratio, as seen in Fig. 10. There was no statistical significance between the LF/HF ratio and any 2D echocardiographic data or the GLS. However, Spearman’s correlation coefficient analysis revealed a positive correlation between LF/HF ratio and GLS, as illustrated in Fig. 11.

## Discussion

Our study included fifty consecutive symptomatic patients with a history of mild to moderate COVID-19 illness after four weeks up to 3 months from the symptom onset with positive PCR results. Severe and critically ill patients were excluded as well as patients who were admitted to the ICU or needed non-invasive or invasive ventilation.

### Prevalence of post-COVID symptoms in the study population

We found that the most common symptom was fatigue followed by dyspnea, then palpitations. With the least reportesd symptoms chest pain and dizziness respectively.

Like our study, *Pavli *et al*.* reported that fatigue was the most commonly experienced symptom, with an incidence ranging from 17.5 to 63%. The second most common symptom was dyspnea and exercise intolerance, with an incidence rate of 10 to 40%. Chest pain affected up to 22% of patients [14].

Moreover, a systemic review held by *Alhumayn *et al*.* revealed similar results. Fatigue and sleep disturbances were reported in two-thirds of the patients, with an incidence of 73% and 85%, respectively, followed by dyspnea, with an incidence of 64% [15].

The main findings in our study are as follows (1) impaired LVGLS was detected in one-fifth of the symptomatic post-COVID patients; however, they had normal baseline LV systolic functions. (2) HRV data analysis using time and frequency domains showed that autonomic dysfunction was prevalent, as 24% of the patients had decreased SDNN. This autonomic dysfunction was related to decreased parasympathetic activity, as there was a higher rMSSD < 40 ms prevalence and LF/HF ratio > 2 in the study population. (3) There was a significant correlation between impaired SDNN and impaired GLS as patients with decreased SDNN had lower LVGLS -15.74 ± 2.61% compared to those with normal SDNN with LVGLS -18.81 ± 1.95.

#### Prevalence of impaired GLS in the study population:

LVGLS was significantly impaired in elderly patients and those with DM&HTN in our post-COVID cohort. This could be explained by the relation between LVGLS impairment, and these factors irrelevant to COVID-19 or that COVID-19-induced subtle LV dysfunction was more evident in these subgroups.

Consistent with the data in the current study, *Mahajan *et al*. reported that Impaired LVGLS was recorded in 1* (*7.7%*)*, 8 *(*13.1%*)*, and 22 *(*44%*)* subjects with asymptomatic, mild, and moderate COVID-19 illness respectively. They concluded that one-third of the study population had impaired GLS. However, in contrast to our study, Mahajan included patients with severe COVID-19 illness, and 99% of the ten patients had impaired GLS, thus significantly contributing to the high incidence of impaired GLS among this study group *[16]*.*

*In addition, Özer *et al*. found that one of third of the patients had impaired LVGLS after one month of acute COVID-19 illness using a cut-off value of* < *− 18%. Seventy-four out of 124 patients were selected after applying the exclusion criteria *[17]*.*

*Turan *et al*.* found that the absolute value of LVGLS was significantly lower in the COVID-19 group than in healthy controls (19.17 ± 2.65 vs. 20.07 ± 2.19, p = 0.03) [18].

Contrary to the data in our study, LVGLS was found to be normal by *Caiado *et al*.,* who conducted a case–control study of 100 post-COVID patients. However, there was an affection of the basal segments' longitudinal strain with a mean of − 16.48 ± 5.41%. Most of them were asymptomatic and mildly symptoms (83%), five patients only had moderate symptoms needing hospitalization, and only 46% of the enrolled populations had post-COVID symptoms [19].

The underlying mechanisms of impaired GLS in post-COVID patients may be attributed to the following mechanisms:- First, direct myocardial cell injury caused by direct viral invasion as described in *Lindner *et al*. *[20] research, which identified the SARS-COV2 virus in the myocardium of COVID-19 patients after autopsy. ACE2 receptor-mediated myocardial injury and an increase in cardiometabolic demand due to systemic infection and hypoxia can also be suggested. Second, systemic inflammation and catecholamines release can cause plaque rupture in atherosclerotic coronaries and increase thrombosis liability in COVID-19 patients. Third, the cytokine storms with the surge of numerous interleukins (e.g., IL 2,6,8,10) and tumor necrosis factor (TNF) can cause myocardial injury. Fourth, the medications used in the Egyptian COVID-19 treatment protocol, such as corticosteroids, antivirals, and immune suppressive agents, can cause myocardial damage. [21–24].

#### HRV data analysis in the study population

In our study, autonomic dysfunction was prevalent as time domain indices SDNN was decreased in 24%, and rMSSD was > 40 ms in 42% of the population, while the frequency domain indices expressed in our study as LF/HF ratio was > 2 in 42% of the patients indicating sympathetic predominance.

In alignment with the data in the current study, *Kurtoğlu *et al*.* stated similar results. *He* conducted a case–control study of 50 patients after mild to moderate COVID-19 with no history of hospitalization, oxygen therapy, or severe respiratory or other significant organ involvement. They found that SDNN, SDANN, and SDNN index were significantly lower in the study group compared to the control group (p < 0.0001 for all). Also, rMSSD was depressed in the study group with a p-value = 0.001. Only the HF band in nu was depressed in the study group compared to the control group. *Kurtoğlu *et al*.* proposed three potential mechanisms for the sympathetic overdominance post-COVID. First, due to the effect of the constitutional symptoms of the acute COVID illness (e.g., fever, sleep disorders, sweating …. etc.). Second, brainstem or medullary mediated increase in sympathetic firing through the binding of the virus to ACE2 receptors found in the glial cells and the neurons. And finally, toxin or immune-mediated mechanism [25].

In opposition to our research, there was an increase in HRV explained by the parasympathetic predominance in COVID-19 patients enrolled in *Kaliyaperumal *et al*. *[26] study. However, patients included in the study were in active COVID-19 illness, unlike our research which mainly targeted the post-COVID population**.**

#### Correlation between GLS and HRV in the study group

Our research pointed out an association between impaired GLS and decreased SDNN. This could be explained as a mere co-existence of both phenomena in post-COVID patients or autonomic dysfunction-induced myocardial affection as proposed by Snelder et al. [27] in a sub-analysis of the CARDIOBESE study. They elaborated that the SDNN index and GLS were not only impaired in obese patients, but also, there was an evident difference in SDNN between obese patients with and without subclinical cardiac dysfunction, and thereby, SDNN was identified as an independent risk factor for cardiac dysfunction. Also the correlation between the GLS and SDNN could be explained by the myocardial injury caused by the cornovirus which may last even after recovery causing subtle LV affection which could be detected by speckle tracking, in addition to this inflammatory response and myocardial injury, the Covid 19 affection can induce sympathetic and parasympathetic imblances causing HRV dysfunction as measured by decreased SDNN.

## Limitations

The small number of patients included and being a single hospital-based study precluded the generalization of the results to all COVID-19 patients. The absence of follow-up was a significant limitation of the current study. The study subjects were examined at a point of time “cross-sectional design”, and thus no data about LVGLS and HRV before the COVID-19 acute infection could be obtained. Increased age, DM, hypertension, and smoking may be confounding factors in our study that may have affected the GLS and HRV parameters' values.

## Conclusion

In patients with prolonged symptoms after COVID-19 illness, GLS and HRV were assessed to try to explain the cardiac involvement. We found a high incidence of GLS impairment using STE and a significant prevalence of diminished HRV. HRV (especially SDNN) and GLS were significantly correlated, as 80% of the patients with impaired GLS had decreased SDNN (p-value < 0.01).
